# Longitudinal effect of HCV cure on markers of kidney disease

**DOI:** 10.1371/journal.pone.0325699

**Published:** 2025-06-11

**Authors:** Javier Cepeda, Mohamed Atta, Katie Zook, Miles Landry, Paula Maier, George Schwartz, Gregory Lucas

**Affiliations:** 1 Department of Epidemiology, Johns Hopkins Bloomberg School of Public Health, Baltimore, Maryland, United States of America; 2 Department of Medicine, Johns Hopkins University School of Medicine, Baltimore, Maryland, United States of America; 3 Department of Pediatrics, University of Rochester Medical Center, Rochester, New York, United States of America; National Taiwan University Hospital, TAIWAN

## Abstract

Chronic hepatitis C infection has been linked to chronic kidney disease. Despite availability of all oral highly curative direct acting antiviral treatment for more than a decade, impact of HCV cure on extrinsically measured iohexol glomerular filtration rate (iGFR), a marker of kidney function, has not been rigorously evaluated. Over two recruitment periods (October 14, 2010 – July 23, 2012 and December 15, 2015 – September 12, 2019), we enrolled 208 participants with chronic HCV infection, 63% of whom were co-infected with HIV. We conducted linear mixed effects modeling to evaluate the change in iGFR slope among participants who were and were not cured from chronic HCV. Secondary outcomes included albuminuria (urine albumin-creatinine-ratio ≥30 mg/g). At baseline, the median age was 51 years (interquartile range: 47–56), most of whom were Black (85%), and male (71%). In the multivariable-adjusted model, including baseline iGFR and other covariates, the adjust difference in iGFR slope was 2.37 mL/min/1.73 m^2^ per year (95% CI: 0.72, 4.03, p = 0.0051) higher among HCV treated participants compared to untreated. HCV treatment status was not associated with probability of albuminuria. Among participants chronically infected with HCV, we identified a significant positive impact of HCV cure on kidney function over time. While iGFR declined overall, declines were attenuated among participants treated for HCV compared to participants who remained untreated.

## Introduction

Chronic HCV is associated with chronic kidney disease (CKD) and end stage renal disease (ESRD). Some studies have shown that patients treated with highly curative direct acting antiviral therapy for chronic HCV infection had reduced kidney disease progression, however others have shown no significant effect between patients who were and were not treated [1–3]. While most studies evaluating the effect of HCV sustained virologic response (SVR) on kidney disease used estimated glomerular filtration rate (eGFR), to our knowledge, none have examined the impact of HCV cure using exogenously measured GFR with iohexol (iGFR). Exogenously measured GFR avoids bias that might arise from non-GFR effects of intrinsic GFR biomarkers. For example, diet, inflammation, and obesity could lead to spurious associations between kidney function and eGFR biomarkers which would not be relevant for iGFR [4].

As availability of all-oral, short-course, interferon-free direct-acting agents (DAAs) for HCV has increased over the past decade, the objective of this analysis was to characterize the natural history and long-term impact of SVR on markers of kidney function and damage in a cohort of participants that were HCV-monoinfected or HCV/HIV coinfected who were eligible for treatment.

## Materials and Methods

### Recruitment and enrollment procedures

In the initial recruitment period of the “Mr. Bean” study (October 14, 2010 – July 23, 2012), we enrolled 92 HIV-positive participants from the Johns Hopkins HIV clinic who were coinfected with HCV and 23 HCV monoinfected participants from a longitudinal study of people who inject drugs. During the second recruitment period (December 15, 2015–September 12, 2019), we enrolled an additional 93 HCV-infected participants (39 HIV-positive and 54 HIV-negative). We focused on HCV-infected participants in the second recruitment period in accordance with a new aim to assess longitudinal effects of HCV cure in the cohort. As this study was not a clinical trial but rather observational, the goal was to characterize the natural history of HCV cure on participants who were eligible to receive treatment. The study did not provide HCV treatment, although we referred HCV-infected individuals to care. Inclusion criteria for this study included being at least 18 years old, estimated GFR ≥ 60 mL min 1.73 m^2^ by the Modiﬁcation of Diet in Renal Disease equation (this threshold was lowered to ≥ 45 mL min 1.73 m^2^ in the second recruitment) and chronically infected with HCV. We excluded participants with a history of radiocontrast allergy, insufﬁcient venous access to place two peripheral intravenous catheters, pregnancy, uncontrolled blood pressure, collagen vascular disease, or severe or life-threating comorbid conditions. We also excluded participants with diabetes to rule out kidney function decline attributed to diabetes rather than HIV or chronic HCV infection.

### Ethics statement

This study was approved by the Johns Hopkins Medicine Institutional Review Board. All participants provided written informed consent.

### Laboratory procedures

At baseline and annual follow-up visits, participants completed an interviewer-administered questionnaire on demographic and behavioral data. We also collected medication data at each visit, based on both electronic medical record review and self-report. We measured iGFR by infusing a weighed amount of iohexol via intravenous catheter, four timed blood draws through a second intravenous catheter, measurement of iohexol plasma concentration in each sample with high performance liquid chromatography, and application of a 2-compartment model to calculate iGFR [5].

We conducted blood testing to assess HIV and HCV serostatus and viral load. HIV RNA was measured with the Amplicor HIV-1 MONITOR Test v1.5 (Roche Molecular Diagnostics, Pleasanton, CA) or RealTime HIV-1 (Abbott Laboratories, Abbott Park, IL). We considered suppressed viral load to be HIV RNA values less than 400 copies per milliliter. HCV infection was deﬁned as positive HCV serology with a detectable HCV RNA at enrollment. For the first recruitment period, HCV RNA testing was conducted using the HCV Ortho Eliza kit assay (Ortho-Clinical Diagnostics, Inc). For the second recruitment period, real-time testing for HCV RNA was sent by Johns Hopkins Medical Laboratories to Quest Diagnostics and completed via real-time polymerase chain reaction. HCV genotyping was conducted by the Johns Hopkins Medical Laboratories. Creatinine was measured with an enzymatic assay (Creatinine Plus, Roche Diagnostics, Basel, Switzerland) that was traceable to an isotope dilution mass spectrometry reference method. Cystatin C was measured using a particle-enhanced turbidimetric immunoassay (Gentian, AS, Norway), with values standardized to certified reference material [6]. Estimated GFR (eGFR) values using creatinine and cystatin c were derived from the 2021 Chronic Kidney Disease Epidemiology Collaboration (CKD-EPI) that excluded a term for race [7]. Urine specimens were collected to determine the albumin-to-creatinine ratio.

### Assessments

#### Missing data.

An individual might have missed their iGFR measurement due to technical challenges of drawing blood versus a participant who missed a visit completely (but returned at a subsequent visit) versus a participant who dropped out of the study completely. Comparisons between participants who were and were not retained after one study visit are presented in Supplementary S1 Table. Further, Supplementary S2 Table, shows the number of valid iGFR, creatinine and cystatin c measurements in the study sample. We also include the number and proportion missing stratified by year, over the entire 8-year observation period.

#### Independent variable.

The primary exposure of interest was HCV infection status (chronic or SVR). Only participants with detectable HCV RNA at the baseline visit (n = 208) were included. We excluded two participants who had been successfully treated with interferon-based HCV treatment prior to enrollment in the study. At follow-up visits, participants’ HCV status was classified as SVR if they had previous chronic infection with undetectable RNA following HCV treatment.

#### Dependent variables.

Our primary dependent variable was iGFR (in mL/min/1.73 m^2^) collected at annual visits. We also evaluated urine albumin-to-creatinine ratio (uACR), which we dichotomized using an established cutoff (≥30 mg/g abnormal).

#### Covariates.

We considered baseline covariates (sex assigned at birth [male, female], race [Black, non-Black], ever smoked at least 100 cigarettes in life [yes/no], history of hypertension [yes/no], ever injected drugs [yes/no]), and HIV status. Time-dependent covariates that we considered included BMI (normal, underweight, overweight, obese), systolic blood pressure (continuous), diastolic blood pressure (continuous), glycosylated hemoglobin (continuous), and ratio of total cholesterol to HDL cholesterol (continuous). We estimated FIB-4 based on AST, ALT, platelet and age and used established cutoff of >3.25 to indicate advanced fibrosis. Additionally, in lieu of the Child-Pugh score, we also estimated the albumin to bilirubin (ALBI) score ((log_10_ bilirubin × 0.66) + (albumin × −0.085), where bilirubin is measured in μmol/L and albumin in g/L) and used established cutoffs (≤−2.60 = Grade 1, > -2.60 to ≤−1.39 = Grade 2, > -1.39 = Grade 3) [8]. These values were available for 78% (n = 163) of participants at their baseline value during the second recruitment period. We modelled follow-up time according to HCV RNA status. If participants were chronically infected, time was number of years since enrollment. After participants were treated, time was reset to number of years since SVR.

### Statistical analyses

We used Chi-square and Wilcoxon rank sum tests to compare categorical and continuous variables, respectively. After verifying that the iGFR was normally distributed, we then fit a linear regression model with fixed and random effects and an unstructured covariance structure. All multivariable linear mixed effects models were adjusted for baseline and time-dependent covariates, as noted previously. In the primary analysis, we included only observations in which the participants had valid same-day measures of iGFR. As iGFR could not be measured at some study visits, as a sensitivity analysis, we repeated the mixed effects modeling after substituting missing iGFR values with serum creatinine- and cystatin C-based eGFR, if available at the same study visit. In a second sensitivity analysis, we repeated the same modeling however we replaced iGFR measures with eGFR instead. Additionally, in a separate sensitivity analysis, we limited the sample to just participants with at least two visits. FIB-4 was not used in the final model as 15% of the observations were missing either AST, ALT, or platelet value and 22% of participants did not have an available ALBI Score. Thus, we conducted a sensitivity analysis for all visits that included non-missing FIB-4 value and ALBI Score. Finally, we modeled albuminuria using logistic regression with random effects. An alpha of 0.05 was the threshold used to determine significance. All statistical procedures were conducted using SAS v.9.4.

## Results

**Fig 1:**
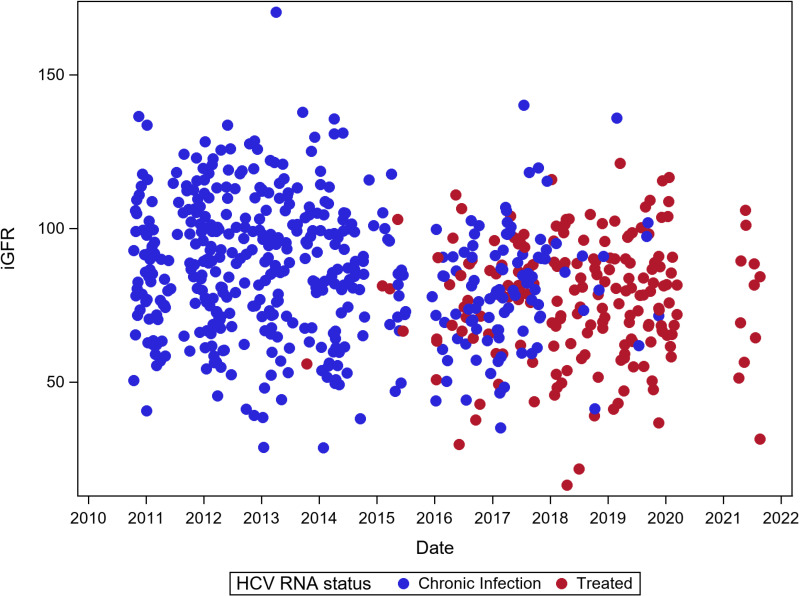
Scatterplot of iGFR measurements over time, stratified by HCV treatment status.

The analytic sample size consisted of 208 HCV-infected participants who provided valid iGFR measurement over 653 study visits. Fig 1 displays the iGFR values over calendar time, stratified by HCV RNA status (chronically infected vs. treated). The median number of visits per participant was 3 (Interquartile Range [IQR]: 1–5). Most participants were male (71%) and Black (85%), with a median age of 51 (IQR: 47–56) at baseline. Among the 77 HCV monoinfected and 131 HCV/HIV dual infected, 45% and 61% respectively, achieved SVR while under observation. The median HCV viral load among participants at their baseline visit was 2,500,000 IU/mL (interquartile range: 642,000 IU/mL, 7,880,000 IU/mL). Approximately 80% had available genotype data (n = 160) and nearly all were genotype 1 (n = 154, 96%). Among participants who either continued to the second wave or were newly enrolled during the second recruitment wave with available information on DAA therapies received (n = 121), 77% were treated with sofosbuvir often in combination with ledipasvir (60%), velpatasvir (12%) or velpatasvir/voxilaprevir (2%). Regimens which included only sofosbuvir accounted for 3% of all treatments received. Most of the remaining regimens included elbasvir/ grazeprevir (9%), glecaprevir/pibrentasvir (7%), and ombitasvir/paritaprevir with or without ribarivin (6%).

When stratified by HIV status, significant differences were observed between HCV monoinfected and HIV/HCV co-infected participants (Table 1). For example, HIV/HCV co-infected participants were less likely to be male yet more likely to be Black. The baseline median iGFR was significantly lower among those with HIV/HCV co-infection compared to HCV monoinfected persons (81 vs. 91 mL/min/1.73m^2^, respectively). However, participants did not differ with respect to non-invasive markers of liver disease (FIB-4 or ALBI Score) by HIV status. Approximately 20% of the sample had FIB-4 > 3.25 and 88% were classified as ALBI Grade 1. Overall, 51 participants (25%) contributed only one study visit. Participants who were not retained for more than one study visit were more likely to be younger, not living with HIV, and had higher iGFR measurement at baseline (see Supplementary S1 Table). Supplementary S3 Table and Supplementary S4 Table show participant characteristics stratified by baseline hematuria and albuminuria, respectively.

**Table 1:** Baseline characteristics of persons monoinfected with HCV or dual infected with HCV/HIV (n = 208).

	HCV monoinfected (n = 77)	HCV/HIV co-infected (n = 131)	p-value
**Male (n, %)**	65 (84)	82 (63)	<0.001
**Black (n, %)**	59 (77)	118 (90)	0.009
**Median (IQR) age**	51.5 (46, 56)	50.6 (47, 56)	0.658
**Smoked at least 100 cigarettes in life (n, %)**	74 (96)	108 (82)	0.004
**Ever injected drugs (n, %)**	64 (83)	97 (74)	0.131
**Hypertension (n, %)**	19 (25)	31 (24)	0.896
**Body mass index (BMI) (n, %)**			0.196
** Underweight**	0 (0)	7 (5)	
** Normal**	37 (48)	54 (41)	
** Overweight**	23 (30)	39 (30)	
** Obese**	17 (22)	31 (24)	
**Glycosylated hemoglobin, %**	5.5 (5.1, 5.7)	5.4 (5.1, 5.7)	0.601
**Systolic blood pressure, mm Hg**	126 (114, 133)	122 (109, 134)	0.270
**Diastolic blood pressure, mm Hg**	74 (68, 82)	72 (66, 80)	0.308
**Total cholesterol, mg/dL**	167 (146, 194)	158 (130, 177)	0.004
**High density lipoprotein, mg/dL**	58 (48, 71)	51 (42, 64)	0.011
**Cystatin C, mg/dL**	1.07 (0.91, 1.22)	1.07 (0.92, 1.26)	0.499
**Creatinine, mg/dL**	0.90 (0.80, 1.00)	0.90 (0.80, 1.10)	0.365
**iohexol glomerular filtration rate (iGFR), mL/min/1.73 m** ^**2**^	91 (79, 106)	81 (70, 98)	0.011
**Estimated glomerular filtration rate (eGFR), mL/min/1.73 m** ^**2**^	87 (76, 98)	80 (66, 94)	0.006
**Alanine aminotransferase(U/L)**	40 (27, 57)	36 (26, 53)	0.558
**Aspartate aminotransferase(U/L)**	44 (33, 67)	46 (31, 65)	0.839
**Platelets (cells/μL)**	224 (178, 276)	207 (167, 247)	0.097
**Fibrosis-4 (FIB-4) > 3.25 (n, %)**	10 (20)	23 (18)	0.709
**Urine albumin creatinine ratio, mg/g**	6 (4, 12)	6 (3, 20)	0.954
**Albumin-Bilirubin Score**	−3.02 (−3.20, −2.78)	−3.01 (−3.27, −2.77)	0.950
**HIV RNA < 400 copies/mL**	----	98 (75)	-----
**CD4 cell count, median (IQR)**	----	440 (247, 662)	-----

**Fig 2:**
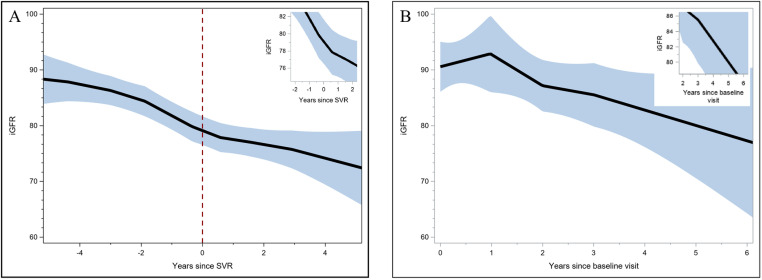
Locally estimated scatterplot smoothing (black line) and 95% confidence intervals (blue band) of iGFR trajectories before and after receipt of DAAs (A) and among participants who were never treated (B) Time scales on x-axes are different.

In participants who were treated during follow-up, the locally estimated scatterplot smoothing function showed a positive inflection in iGFR slope a few months after successful HCV treatment (Fig 2A, inset). In contrast, among people who were not treated for HCV while under observation, there was a change in iGFR slope in the first year of follow-up, followed by a downward trajectory thereafter (Fig 2B). In the unadjusted model for the entire sample, the difference in iGFR slope between HCV treated and untreated participants was 1.94 mL min 1.73 m^2^ (95% Confidence Interval [CI] 0.22, 3.66, p = 0.027 for interaction, Table 2). When stratified by HIV status, the difference in iGFR slope among those who were HCV monoinfected was greater (3.08 mL/min/1.73 m^2^ per year) compared to the co-infected group; however this association was not statistically significant (p = 0.289). In the adjusted model, the estimated slopes were −1.92 mL/min/1.73 m^2^ per year (95% CI: −2.71, −1.13) and 0.45 mL/min/1.73 m^2^ per year (95% CI: −1.03, 1.93) in HCV untreated and treated persons respectively, with HCV treatment associated with a change in slope of 2.37 mL min 1.73 m^2^ per year (95% CI: 0.72, 4.03, p = 0.005 for interaction). This pattern of an upward inflection in iGFR slope was similar in HIV-positive and HIV-negative subgroups, although the interaction was only statistically significant in the HIV-positive stratum. In a sensitivity analysis where we replaced missing iGFR values with eGFR (n = 43), differences in iGFR slope were unchanged (2.67 mL/min/1.73 m^2^ per year for change in slope in treated vs. untreated, 95% CI: 0.95, 4.38, p = 0.002 for interaction). In a second sensitivity analysis where we modeled eGFR, we detected a stronger difference in eGFR slope (4.62 mL/min 1.73 m^2^ per year for change in slope in HCV treated vs. untreated, 95% CI: 3.02, 6.24, p < .001 for interaction). In sensitivity analyses, limiting the analysis to either participants who had at least two visits and only observations or with non-missing FIB-4 and ALBI values did not substantially change the association between HCV treatment and iGFR over time (Supplementary S5 and S6 Tables).Multivariable mixed effects indicated that there was no significant difference in log odds of albuminuria associated with a change in HCV RNA status (Supplementary S7 Table).

**Table 2:** Estimated iohexol glomerular filtration rate (iGFR) slopes (mL min 1.73 m^2^ per year) in untreated and treated persons with HCV, overall and stratified by HIV status.

Factor	Unadjusted associations		Adjusted associations*	
	Difference in iGFR slope, (linear time interaction)	p-value	Difference in iGFR slope, (linear time interaction)	p-value
**Overall sample**				
** Chronic infection**	Ref		Ref	
** SVR**	1.94 (0.22, 3.66)	0.027	2.37 (0.72, 4.03)	0.005
**HCV monoinfected**				
** Chronic infection**	Ref		Ref	
** SVR**	3.08 (−2.66, 8.83)	0.289	3.20 (−3.24, 9.64)	0.325
**HCV/HIV coinfected**				
** Chronic infection**	Ref		Ref	
** SVR**	1.82 (0.05, 3.59)	0.044	2.62 (0.91, 4.33)	0.003

*Adjusted for: baseline iGFR, sex, race, ever smoked at least 100 packs in life, history of hypertension, ever injected drugs, body mass index (BMI), systolic blood pressure, diastolic blood pressure, glycosylated hemoglobin, and ratio of total cholesterol to high density lipoprotein (HDL) cholesterol.

## Discussion

We found evidence supporting a positive impact of HCV cure on kidney function in HCV monoinfected and HCV/HIV coinfected individuals. While iGFR declined among all participants in the cohort, declines were attenuated among participants successfully treated for HCV compared to participants who remained untreated. In contrast, HCV cure was not significantly associated with changes in albuminuria. [9] Kidney function measured by GFR and albuminuria are the two axes of characterizing chronic kidney disease. [10] In our cohort, baseline albuminuria was low, which might have limited our ability to detect an effect of SVR, unlike the association we observed for iGFR.

Emerging data from other studies has yielded inconsistent results, with studies both finding (Liu and Sise) and not finding (O’Donnell) improved kidney function with SVR [1–3]. Our study is unique in using a gold-standard, extrinsic measure of GFR – iohexol disappearance from plasma. While GFR estimates from intrinsic biomarkers, such as serum creatinine and cystatin C, are convenient and widely used in clinical practice, it is well established that intrinsic markers of kidney function are affected by non-GFR factors. This is relevant in assessing changes in kidney function following SVR, which is associated with reductions in inflammatory markers. [4] Previously we found that both HCV infection and non-suppressed HIV RNA (among HIV-positive persons) were associated with lower eGFR accuracy and larger bias, compared with HCV-uninfected and HIV RNA suppressed status, respectively [5]. As HIV is also independently associated with kidney disease progression, our findings are consistent by demonstrating HIV positivity to be associated with lower mean iGFR values. HCV is an independent risk factor for CKD in both HIV-positive and HIV-negative persons. Although our study did not include kidney biopsies, it has long been recognized that HIV and HCV are associated with distinct pathologic kidney diseases, HIV-associated nephropathy and immune complex glomerulonephritis, respectively [11,12]. However, these specific pathologic entities only account for a fraction of common CKD seen in clinical practice.

Our study is subject to several limitations. This study might not be generalizable to other settings. Additionally, participants who were not treated for HCV while under observation may have different care from those who were treated in ways not captured by multivariable adjustment. Thus, the association between HCV treatment and improvement in kidney disease might also be partially explained by engagement in care which could have also affected management of chronic conditions. Additionally, due to the relatively few HCV-monoinfected participants, we did not observe a statistically significant interaction with treatment status on iGFR, as opposed to the association observed among HIV/HCV co-infected participants. Finally, approximately 25% of the sample contributed only one iGFR measurement, which could have limited robustness of findings. Strengths of the study include a prospective cohort design, gold-standard GFR measurement, standardized data collection methods across a decade, and inclusion of both HCV monoinfected and HCV/HIV coinfected participants. In conclusion, our findings lend further evidence on the potential positive impact of HCV cure on reducing kidney disease progression in patients with and without HIV co-infection. In conclusion, our findings lend further evidence on the potential positive impact of HCV cure on reducing kidney disease progression in patients with and without HIV co-infection.

## Supporting information

S1 Table Baseline characteristics of persons who contributed only one iGFR visit versus those who contributed more than one visit. (DOCX)

S2 Table Number of valid and missing iGFR, serum creatinine, and serum cystatin c measurements by study visit. (DOCX)

S3 Table Baseline characteristics of persons with and without albuminuria (uACR ≥ 30 mg/g) at baseline. (DOCX)

S4 Table Baseline characteristics of persons with and without hematuria at baseline. (DOCX)

S5 Table Estimated iGFR slopes (mL min 1.73 m2 per year) in untreated and treated persons with HCV, overall and stratified by HIV status including with at least two iGFR visits. (DOCX)

S6 Table Estimated iGFR slopes (mL min 1.73 m2 per year) in untreated and treated persons with HCV, overall and stratified by HIV status including FIB-4 and ALBI grade. (DOCX)

S7 Table Adjusted associations of albuminuria (uACR ≥ 30 mg/g). (DOCX)
