# Supplementary material for: Longitudinal effect of HCV cure on markers of kidney disease
Source: PLoS One. 2025 Jun 11;20(6):e0325699. doi: 10.1371/journal.pone.0325699 (PMC12157062; doi:10.1371/journal.pone.0325699)
Supplement: S1 Table — (DOCX) [file pone.0325699.s001.docx]

**Table S1: Baseline characteristics of persons who contributed only one iohexol glomerular filtration rate (iGFR) visit versus those who contributed more than one visit.**

|  | **Contributed only one visit (n=51)** | **Contributed more than one visit (n=157)** | **p-value** |
| --- | --- | --- | --- |
| **Male** | 41 (80) | 106 (68) | 0.0793 |
| **Black** | 39 (76) | 138 (88) | 0.0465 |
| **Median (IQR) age** | 50 (46 – 56) | 56 (52 – 61) | <.0001 |
| **Smoked at least 100 cigarettes in life** | 45 (88) | 137 (87) | 0.8550 |
| **Ever injected drugs** | 44 (86) | 117 (75) | 0.0812 |
| **HIV positive** | 24 (47) | 107 (68) | 0.0067 |
| **Hypertension (yes)** | 12 (24) | 38 (24) | 0.9220 |
| **Body mass index (BMI)**  **Underweight**  **Normal**  **Overweight**  **Obese** | 1 (2)  28 (55)  13 (25)  9 (18) | 6 (4)  63 (40)  49 (31)  39 (25) | 0.3077 |
| **Glycosylated hemoglobin, %** | 5.5 (5.1 – 5.7) | 5.6 (5.3 – 5.8) | 0.1119 |
| **Systolic blood pressure, mm Hg** | 122 (113 – 134) | 124 (111 – 133) | 0.7652 |
| **Diastolic blood pressure, mm Hg** | 73 (68 – 81) | 72 (66 – 80) | 0.6288 |
| **Total cholesterol, mg/dL** | 154 (133 – 191) | 164 (144 – 186) | 0.1572 |
| **High density lipoprotein , mg/dL** | 52 (39 – 76) | 50 (42-65) | 0.4420 |
| **Cystatin C, mg/dL** | 1.13 (0.99 – 1.30) | 1.07 (0.96 – 1.27) | 0.4138 |
| **Creatinine, mg/dL** | 0.90 (0.80 – 1.10) | 1.00 (0.90 – 1.20) | 0.0654 |
| **iohexol glomerular filtration rate (iGFR), mL/min /1.73 m^2^** | 90 (73 – 102) | 81 (67 – 96) | 0.0354 |
| **Estimated glomerular filtration rate (eGFR), mL/min /1.73 m^2^** | 83 (72 – 94) | 86 (68 – 101) | 0.5754 |
| **Urine albumin creatinine ratio, mg/g** | 6 (3-21) | 8 (4-22) | 0.2922 |
