# Supplementary material for: Longitudinal effect of HCV cure on markers of kidney disease
Source: PLoS One. 2025 Jun 11;20(6):e0325699. doi: 10.1371/journal.pone.0325699 (PMC12157062; doi:10.1371/journal.pone.0325699)
Supplement: S2 Table — (DOCX) [file pone.0325699.s002.docx]

**Table S2. Number of valid and missing iohexol glomerular filtration rate (iGFR), serum creatinine, and serum cystatin c measurements by study visit. Note: Visit 1 is baseline for the first cohort recruited from October, 2010 – July, 2012 and Visit 5 is the baseline for the second cohort recruited from December, 2015- October, 2019.**

|  | Visit 1 | Visit 2 | Visit 3 | Visit 4 | Visit 5 | Visit 6 | Visit 7 | Visit 8 |
| --- | --- | --- | --- | --- | --- | --- | --- | --- |
| iGFR | 112 | 91 | 82 | 78 | 132 | 80 | 56 | 22 |
| Missing iGFR (% of total) | 2 (1.7%) | 1 (1.1%) | 0 (0%) | 0 (0%) | 41 (23.7%) | 30 (27.2%) | 11 (16.4%) | 3 (12.0%) |
| Creatinine | 114 | 91 | 81 | 78 | 167 | 102 | 63 | 25 |
| Missing creatinine (% of total) | 0 (0%) | 1 (1.1%) | 1 (1.2%) | 0 (0%) | 6 (3.5%) | 8 (7.8%) | 4 (6.0%) | 0 (0%) |
| Cystatin C | 114 | 88 | 81 | 77 | 166 | 103 | 64 | 25 |
| Missing Cystatin c (% of total) | 0 (0%) | 4 (4.3%) | 1 (1.2%) | 1 (1.3%) | 7 (4.0%) | 7 (6.4%) | 3 (4.5%) | 0 (0%) |
