# Supplementary material for: Longitudinal effect of HCV cure on markers of kidney disease
Source: PLoS One. 2025 Jun 11;20(6):e0325699. doi: 10.1371/journal.pone.0325699 (PMC12157062; doi:10.1371/journal.pone.0325699)
Supplement: S3 Table — (DOCX) [file pone.0325699.s003.docx]

**Table S3: Baseline characteristics of persons with and without albuminuria (urine albumin creatinine ratio ≥30 mg/g) at baseline**

|  | **Urine albumin creatinine ratio ≥30 mg/g**  **(n=32)** | **Urine albumin creatinine ratio <30 mg/g**  **(n=176)** | **p-value** |
| --- | --- | --- | --- |
| **Male** | 18 (56) | 129 (73) | 0.05 |
| **Black** | 32 (100) | 145 (82) | 0.01 |
| **Median (IQR) age** | 54 (51 – 59) | 51 (46 – 55) | 0.001 |
| **Smoked at least 100 cigarettes in life** | 27 (84) | 155 (88) | 0.56 |
| **Ever injected drugs** | 24 (75) | 137 (78) | 0.72 |
| **Hypertension (yes)** | 11 (34) | 39 (22) | 0.14 |
| **Body mass index (BMI)**  **Underweight**  **Normal**  **Overweight**  **Obese** | 1 (3)  16 (50)  8 (25)  7 (22) | 6 (3)  75 (43)  54 (31)  41 (23) | 0.88 |
| **Glycosylated hemoglobin, %** | 5.4 (5.2 – 5.7) | 5.4 (5.1 – 5.7) | 0.58 |
| **Systolic blood pressure, mm Hg** | 131 (123 – 148) | 122 (111 – 132) | 0.005 |
| **Diastolic blood pressure, mm Hg** | 76 (71 – 88) | 72 (66 – 80) | 0.007 |
| **Total cholesterol, mg/dL** | 167 (138 – 188) | 161 (138 – 189) | 0.90 |
| **High density lipoprotein, mg/dL** | 51(37 – 64) | 53 (45 – 68) | 0.20 |
| **Cystatin C, mg/dL** | 1.18 (0.94 – 1.34) | 1.06 (0.90 – 1.22) | 0.05 |
| **Creatinine, mg/dL** | 0.90 (0.80 – 1.10) | 0.90 (0.80 – 1.10) | 0.84 |
| **iohexol glomerular filtration rate (iGFR), mL/min /1.73 m^2^** | 77 (70 – 92) | 89 (74 – 101) | 0.06 |
| **estimated glomerular filtration rate (eGFR), mL/min /1.73 m^2^** | 75 (63 – 94) | 86 (72 – 97) | 0.02 |
| **HIV positive** | 23 (72) | 106 (61) | 0.26 |
