# Supplementary material for: Longitudinal effect of HCV cure on markers of kidney disease
Source: PLoS One. 2025 Jun 11;20(6):e0325699. doi: 10.1371/journal.pone.0325699 (PMC12157062; doi:10.1371/journal.pone.0325699)
Supplement: S4 Table — (DOCX) [file pone.0325699.s004.docx]

**Table S4: Baseline characteristics of persons with and without hematuria at baseline**

|  | **Hematuria**  **(n=19)** | **No hematuria**  **(n=187)** | **p-value** |
| --- | --- | --- | --- |
| **Male** | 12 (63) | 134 (72) | 0.44 |
| **Black** | 16 (84) | 159 (85) | 0.92 |
| **Median (IQR) age** | 50 (45 – 55) | 51 (47 – 56) | 0.81 |
| **Smoked at least 100 cigarettes in life** | 2 (8) | 17 (9) | 0.77 |
| **Ever injected drugs** | 5 (11) | 14 (9) | 0.62 |
| **Hypertension (yes)** | 4 (21) | 46 (25) | 0.73 |
| **Body mass index (BMI)**  **Underweight**  **Normal**  **Overweight**  **Obese** | 1 (5)  9 (47)  6 (32)  3 (16) | 6 (3)  81 (43)  55 (29)  45 (24) | 0.85 |
| **Glycosylated hemoglobin, %** | 5.2 (4.9 – 5.6) | 5.4 (5.1 – 5.7) | 0.22 |
| **Systolic blood pressure, mm Hg** | 127 (117 – 141) | 123 (112 – 133) | 0.29 |
| **Diastolic blood pressure, mm Hg** | 74 (65 – 77) | 73 (66 – 81) | 0.97 |
| **Total cholesterol, mg/dL** | 155 (124 – 192) | 161 (139 – 186) | 0.61 |
| **High density lipoprotein, mg/dL** | 54 (43 – 70) | 52 (44 – 68) | 0.92 |
| **Cystatin C, mg/dL** | 1.12 (0.93 – 1.23) | 1.07 (0.91 – 1.25) | 0.53 |
| **Creatinine, mg/dL** | 0.90 (0.70 – 1.20) | 0.90 (0.80 – 1.10) | 0.88 |
| **iohexol glomerular filtration rate (iGFR), mL/min /1.73 m^2^** | 85 (75 – 101) | 86 (73 – 100) | 0.96 |
| **estimated glomerular filtration rate (eGFR), mL/min /1.73 m^2^** | 85 (65 – 94) | 83 (80 – 96) | 0.63 |
| **Urine albumin creatinine ratio, mg/g** | 22 (6 – 65) | 6 (3 – 13) | 0.004 |
| **HIV positive** | 7 (9) | 12 (9) | 1.00 |
