# Supplementary material for: Longitudinal effect of HCV cure on markers of kidney disease
Source: PLoS One. 2025 Jun 11;20(6):e0325699. doi: 10.1371/journal.pone.0325699 (PMC12157062; doi:10.1371/journal.pone.0325699)
Supplement: S5 Table — (DOCX) [file pone.0325699.s005.docx]

**Table S5. Estimated iohexol glomerular filtration rate (iGFR)slopes (mL min 1.73 m^2^ per year**) **in untreated and treated persons with HCV, overall and stratified by HIV status including with at least two visits**

|  | **Unadjusted associations** | | **Adjusted associations*** | |
| --- | --- | --- | --- | --- |
| **Factor** | **Difference in iGFR slope, (linear time interaction)** | **p-value** | **Difference in iGFR slope, (linear time interaction)** | **p-value** |
| **Overall sample**  **Chronic infection**  **SVR** | Ref  1.98 (0.26, 3.71) | 0.024 | Ref  2.43 (0.72, 4.14) | 0.006 |
| **HCV monoinfected**  **Chronic infection**  **SVR** | Ref  3.07 (-2.71, 8.85) | 0.293 | Ref  3.65 (-3.39, 10.70) | 0.304 |
| **HCV/HIV coinfected**  **Chronic infection**  **SVR** | Ref  1.92 (0.15, 3.70) | 0.034 | Ref  2.70 (0.95, 4.42) | 0.003 |

***Adjusted for: baseline iohexol glomerular filtration rate (iGFR), sex, race, ever smoked at least 100 packs in life, history of hypertension, ever injected drugs, body mass index (BMI), systolic blood pressure, diastolic blood pressure, glycosylated hemoglobin, and ratio of total cholesterol to high-density lipoprotein (HDL) cholesterol.**
