# Supplementary material for: Longitudinal effect of HCV cure on markers of kidney disease
Source: PLoS One. 2025 Jun 11;20(6):e0325699. doi: 10.1371/journal.pone.0325699 (PMC12157062; doi:10.1371/journal.pone.0325699)
Supplement: S6 Table — (DOCX) [file pone.0325699.s006.docx]

**Table S6. Estimated iohexol glomerular filtration rate (iGFR)slopes (mL min 1.73 m^2^ per year**) **in untreated and treated persons with HCV, overall and stratified by HIV status including fibrosis-4 (FIB-4) and albumin bilirubin (ALBI) grade**

|  | **Unadjusted associations** | | **Adjusted associations*** | |
| --- | --- | --- | --- | --- |
| **Factor** | **Difference in iGFR slope, (linear time interaction)** | **p-value** | **Difference in iGFR slope, (linear time interaction)** | **p-value** |
| **Overall sample**  **Chronic infection**  **SVR** | Ref  1.94 (0.22, 3.66) | 0.027 | Ref  1.95 (0.06, 3.83) | 0.043 |
| **HCV monoinfected**  **Chronic infection**  **SVR** | Ref  3.08 (-2.66, 8.83) | 0.289 | Ref  1.38 (-9.30, 12.06) | 0.738 |
| **HCV/HIV coinfected**  **Chronic infection**  **SVR** | Ref  1.82 (0.05, 3.59) | 0.044 | Ref  2.42 (0.48, 4.35) | 0.015 |

***Adjusted for: baseline iohexol glomerular filtration rate (iGFR), sex, race, ever smoked at least 100 packs in life, history of hypertension, ever injected drugs, body mass index (BMI), fibrosis-4 (FIB-4) >3.25, albumin bilirubin (ALBI) grade, systolic blood pressure, diastolic blood pressure, glycosylated hemoglobin, and ratio of total cholesterol to high-density lipoprotein (HDL) cholesterol.**
