# Supplementary material for: Longitudinal effect of HCV cure on markers of kidney disease
Source: PLoS One. 2025 Jun 11;20(6):e0325699. doi: 10.1371/journal.pone.0325699 (PMC12157062; doi:10.1371/journal.pone.0325699)
Supplement: S7 Table — (DOCX) [file pone.0325699.s007.docx]

**Table S7: Adjusted associations of albuminuria (uACR≥30 mg/g)**

|  | **Unadjusted association and interaction with time** | | **Adjusted association and interaction with time*** | |
| --- | --- | --- | --- | --- |
| **Factor** | **Difference in log odds of albuminuria** | **p-value** | **Difference in log odds of albuminuria** | **p-value** |
| **Overall sample**  **Chronic infection**  **SVR** | Ref  -0.41 (-0.85, 0.02) | 0.0645 | Ref  -0.38 (-0.86, 0.10) | 0.1243 |
| **HCV RNA monoinfected**  **Chronic infection**  **SVR** | Ref  -0.69 (-6.20, 4.82) | 0.8043 | Ref  0.74 (-5.64, 7.12) | 0.8178 |
| **HCV RNA/HIV coinfected**  **Chronic infection**  **SVR** | Ref  -0.32 (-0.73, 0.09) | 0.1289 | Ref  -0.36 (0.83, 0.11) | 0.1298 |

***Adjusted for: sex, race, ever smoked at least 100 packs in life, history of hypertension, ever injected drugs, body mass index (BMI), systolic blood pressure, diastolic blood pressure, glycosylated hemoglobin, and ratio of total cholesterol to high-density lipoprotein (HDL) cholesterol.**
